# Metabolic Abnormalities Detected in Phase II Evaluation of Doxycycline in Dogs with Multicentric B-Cell Lymphoma

**DOI:** 10.3389/fvets.2018.00025

**Published:** 2018-02-26

**Authors:** Kelly R. Hume, Skylar R. Sylvester, Lucia Borlle, Cheryl E. Balkman, Angela L. McCleary-Wheeler, Mary Pulvino, Carla Casulo, Jiyong Zhao

**Affiliations:** ^1^Department of Clinical Sciences, College of Veterinary Medicine, Cornell University, Ithaca, NY, United States; ^2^College of Veterinary Medicine, Cornell University, Ithaca, NY, United States; ^3^Department of Biomedical Genetics, University of Rochester Medical Center, Rochester, NY, United States; ^4^Wilmot Cancer Institute, University of Rochester Medical Center, Rochester, NY, United States

**Keywords:** dogs, hematopoietic neoplasm, tetracycline toxicity, doxycycline, lymphoma

## Abstract

Doxycycline has antiproliferative effects in human lymphoma cells and in murine xenografts. We hypothesized that doxycycline would decrease canine lymphoma cell viability and prospectively evaluated its clinical tolerability in client-owned dogs with spontaneous, nodal, multicentric, substage a, B-cell lymphoma, not previously treated with chemotherapy. Treatment duration ranged from 1 to 8 weeks (median and mean, 3 weeks). Dogs were treated with either 10 (*n* = 6) or 7.5 (*n* = 7) mg/kg by mouth twice daily. One dog had a stable disease for 6 weeks. No complete or partial tumor responses were observed. Five dogs developed grade 3 and/or 4 metabolic abnormalities suggestive of hepatopathy with elevations in bilirubin, ALT, ALP, and/or AST. To evaluate the absorption of oral doxycycline in our study population, serum concentrations in 10 treated dogs were determined using liquid chromatography tandem mass spectrometry. Serum levels were variable and ranged from 3.6 to 16.6 µg/ml (median, 7.6 µg/ml; mean, 8.8 µg/ml). To evaluate the effect of doxycycline on canine lymphoma cell viability *in vitro*, trypan blue exclusion assay was performed on canine B-cell lymphoma cell lines (17-71 and CLBL) and primary B-cell lymphoma cells from the nodal tissue of four dogs. A doxycycline concentration of 6 µg/ml decreased canine lymphoma cell viability by 80%, compared to matched, untreated, control cells (mixed model analysis, *p* < 0.0001; Wilcoxon signed rank test, *p* = 0.0313). Although the short-term administration of oral doxycycline is not associated with the remission of canine lymphoma, combination therapy may be worthwhile if future research determines that doxycycline can alter cell survival pathways in canine lymphoma cells. Due to the potential for metabolic abnormalities, close monitoring is recommended with the use of this drug in tumor-bearing dogs. Additional research is needed to assess the tolerability of chronic doxycycline therapy.

## Introduction

Lymphoma is a malignant cancer of the lymphocytes. While it can arise in almost any organ, it occurs most often in the lymphoid tissues such as lymph nodes, spleen, and liver (1). It is a common neoplasm in both dogs and people (2, 3). Aggressive, diffuse large B-cell lymphoma (DLBCL) is the most frequently diagnosed subtype in both species (3, 4). In dogs, the classification of lymphoma is based on clinical presentation, cell morphology, grade, and immunophenotype, with some controversy over the most appropriate classification scheme (4–6). Molecular profiling has shown the heterogeneity of DLBCL in dogs (7), although the ability to perform such analysis is not yet possible in routine clinical practice. Routine cytology and flow cytometry are often used to diagnose lymphoma. There are some limitations, however, in using these methods to identify less common, indolent subtypes (7–9). Treatment recommendations for canine lymphoma are generally based on whether the disease is indolent or aggressive and whether specific non-lymphoid organs are involved. To date, the most commonly recommended treatment regimen is a multidrug chemotherapy protocol with cyclophosphamide, doxorubicin (hydroxydaunorubicin), vincristine, and prednisone (CHOP) (8, 10–13). Unfortunately, despite high remission rates, the majority of dogs will relapse and eventually die of their cancer within 1–2 years from the time of diagnosis. For B-cell lymphoma, the median survival times of 300–400 days are typically reported (14–16). CHOP chemotherapy is cost-prohibitive for many dog-owning families, as the use of pet insurance is not currently widespread. The potential for toxicity is also a deterrent in some instances. A variety of novel therapies are in development but either lack randomized controlled trial comparisons to CHOP or are not yet commercially available (17–22).

Doxycycline is a low-cost, widely prescribed oral tetracycline antibiotic used in the treatment of a broad range of microorganisms (23–25). In addition to its antimicrobial properties, doxycycline can inhibit matrix metalloproteinases (MMPs) and thereby have antiangiogenic effects (26–28). MMP9 expression seems particularly important in lymphoma (29). In one study, the expression was increased in 50% of DLBCL and was prognostic for poor survival (30). In dogs with B-cell lymphoma, MMP9 expression has been found to decrease with chemotherapy (31). The inhibition of MMP9 may thus have a therapeutic benefit in lymphoma, and doxycycline may represent a means to achieve this goal. NF-κB is a transcriptional regulator of MMPs, and doxycycline has recently been shown to inhibit NF-κB and other important signaling events in human DLBCL cells (32). Cell survival pathways were also altered, suggesting that additivity or synergy with chemotherapeutics or targeted agents may ultimately be possible. Although the effect may be dependent on dosage, administration route, and/or subtype, doxycycline concentrations of 2–6 µg/ml inhibited the proliferation of human DLBCL cells and xenograft tumors in mice (32, 33). Given that doxycycline concentrations within this range can be achieved in canine sera (34, 35), we hypothesized that doxycycline would have an activity against canine B-cell lymphoma and tested this hypothesis in a phase II clinical trial.

## Materials and Methods

This research was performed in accordance with protocols approved by the Cornell University Institutional Animal Care and Use Committee (IACUC; #2014-0022 and #2005-0151). Written informed consent was obtained from each dog’s owner prior to study enrolment. Study medications, clinical evaluation, and monitoring were performed at no cost to owners.

### Clinical Evaluation

Clinical trial enrolment was offered to owners of dogs with lymphoma that declined either single- or multi-agent cytotoxic chemotherapy treatment for their dog. For inclusion, dogs had to (i) have cytologic evidence of lymphoma in an enlarged peripheral lymph node that was characterized by a monomorphic population of intermediate to large lymphocytes, (ii) be substage a, (iii) not have received prior cytotoxic chemotherapy or corticosteroids as treatment for their lymphoma, and (iv) not have received doxycycline within 7 days of enrolment. Flow-cytometric confirmation of B-cell lymphoma was required for continued participation. All associated clinical procedures were performed through the Cornell University Hospital for Animals (CUHA) Oncology Service. All dogs were diagnosed by a board-certified veterinary clinical pathologist based on the morphologic evaluation of lymph node fine-needle aspirates with subsequent diagnostic confirmation and immunophenotyping *via* flow cytometry. Cytologic and flow-cytometric testing were performed through the Clinical Pathology and Anatomic Pathology Laboratories of the Cornell University Animal Health Diagnostic Center (AHDC), Ithaca, NY. The AHDC and its member laboratories are accredited by the American Association of Veterinary Laboratory Diagnosticians. Dogs underwent diagnostic testing for disease staging at the time of study enrolment, including complete blood count (CBC), serum biochemical analysis, urinalysis, thoracic radiography, and abdominal ultrasonography. The first nine dogs enrolled also had cytologic evaluation of bone marrow aspirates. Cancer stage and substage for all enrolled dogs were determined according to World Health Organization criteria (1).

The clinical trial was designed as a prospective, 8-week, single-stage, phase II trial in order to determine single-agent efficacy of doxycycline in canine B-cell lymphoma. To determine our enrolment target, exact, single-stage, phase II sample size tables were used (36). A target activity or a response rate of 35% was chosen as the low end of accepted response rates in treatment protocols used for refractory or resistant canine lymphoma (37–41). *P*_0_ was chosen as 5% as a purposefully low threshold, given the preliminary nature of this investigation. Using a purposefully low threshold allows investigators to begin planning additional trials (e.g., phase III) once the necessary responses are seen or, alternatively, to halt the trial once it is obvious that the number of desired responses will not be observed. With an effect size of 0.3 (0.35 − 0.05), our enrolment target was 11 dogs. With this sample size, we would have 80% power (α = 0.05) to conclude that drug activity was significantly different from 5% (*p*_0_) if we observed clinical responses in three dogs.

The planned doxycycline treatment dosage was 10 mg/kg PO BID. This dosage is within the range previously evaluated in dogs and was chosen as the maximum that could be prescribed based on the available toxicity data at the time of study design (23, 24, 42). Metoclopramide (0.4–0.5 mg/kg PO BID) was prescribed concurrently in an effort to preemptively mitigate any gastrointestinal toxicity. Dogs did not receive any anticancer therapies such as steroids, chemotherapy, or radiation therapy while receiving doxycycline. Due to unknown interactions, no neutraceutical or herbal supplements were administered to dogs during the study. All owners were instructed not to administer doxycycline with any dairy products.

In order to better understand drug tolerability, four additional dogs with B-cell lymphoma were treated in a separate, 4-week study after enrolment in the phase II trial was halted. These dogs were also diagnosed by morphologic evaluation of lymph node fine-needle aspirates with subsequent diagnostic confirmation and immunophenotyping *via* flow cytometry through the Clinical Pathology Laboratory of the AHDC. For disease staging, these dogs had diagnostic testing that included CBC, serum biochemical analysis, urinalysis, thoracic radiography, and abdominal ultrasonography. (Cytologic evaluation of bone marrow aspirates was not performed.)

In the phase II study (*n* = 9), response to doxycycline was assessed according to criteria defined in the Veterinary Cooperative Oncology Group (VCOG) consensus document for peripheral nodal lymphoma in dogs (43). These guidelines define progressive disease (PD) as ≥20% increase in the mean sum of the longest diameter measurements (mean sum LD) of target lesions. Less stringent criteria for PD were used in the separate cohort of four dogs. For these four dogs, PD was defined as ≥50% increase in the mean sum LD, in accordance with values outlined for human lymphoma clinical trials (44). All dogs were evaluated for response weekly for 4 weeks and then for every other week for the remaining 4 weeks, if applicable. Should PD occur, doxycycline was to be discontinued, and owners would have the option of pursuing any additional treatments recommended by the attending clinician.

Adverse events (AEs) were graded according to the VCOG-Common Terminology Criteria for Adverse Events v1.1 (VCOG-CTCAE) (45). Subjective monitoring was performed by attending clinicians and dog owners. CBC and serum biochemical analysis were initially scheduled for evaluation every 2 weeks, but when metabolic abnormalities consistent with hepatopathy were noted within the first 2 weeks of treatment, the frequency of monitoring was increased to weekly, although some dogs had schedule modifications at the discretion of the attending clinician. Doxycycline was temporarily discontinued in any dog that developed a grade 4 AE and resumed at a lower dosage if/when toxicity improved. Dosage de-escalation was planned if greater than one of six dogs experienced a grade 3 or higher gastrointestinal toxicity or an increase in bilirubin or ALT.

### Measurement of Doxycycline in Dog Sera

Serum was collected after dogs had been receiving doxycycline PO BID for 1 week. To capture peak serum concentration, blood was collected 3 h after doxycycline administration when possible (34, 35, 46). After clot formation, serum was separated *via* centrifugation and then stored at −80°C. Doxycycline concentrations were measured as previously described (32) with minor modifications. Briefly, serum samples (100 µl) from dogs treated with doxycycline were mixed with two volumes (200 µl) of acetonitrile in Eppendorf LoBind tubes and vortexed for 6 min at room temperature. The samples were centrifuged at 20,000 *g* for 10 min. The supernatants were collected and dried down. The dried samples were dissolved in 50% methanol, centrifuged (18,000 *g*, 2 min) to remove any debris, and analyzed by liquid chromatography tandem mass spectrometry (LC-MS/MS) at the University of Rochester Proteomics Center. Samples were analyzed in triplicate, and the mean values reported for each dog. For the preparation of standard curves for calculation, appropriate amounts of doxycycline were spiked with sera from untreated dogs, and the samples were processed as described for the serum samples from the treated dogs.

### *In Vitro* Effect of Doxycycline on Viability of Canine Lymphoma Cells

Prior to the initiation of any therapy, lymphoma cells were sterilely collected *via* fine-needle aspiration or excisional biopsy of an affected peripheral lymph node from four dogs (dogs A, B, C, and D) later confirmed to have B-cell lymphoma (characterized by a monomorphic population of intermediate to large lymphocytes). (Dog C also participated in the phase II study.) Cells were injected into RPMI supplemented with 20% fetal bovine serum (FBS) (Sigma-Aldrich). Cells were maintained at 4^°^C or frozen at −80^°^C and shipped overnight to the University of Rochester Medical Center for cell viability assays. An additional sample from each dog was collected at the same time and submitted to the Cornell University AHDC for cytologic or histologic diagnosis and immunophenotyping *via* flow cytometry. For viability analysis, the samples were further dispersed with Gibco Cell Dissociation Buffer (Thermo Fisher Scientific) with 20% FBS and then washed with the same RPMI medium. The cell suspensions were then loaded over a Ficoll-paque Plus (GE Healthcare Life Sciences) density gradient and centrifuged at 400 *g* for 30 min according to the manufacturer’s instructions. Isolated cells were washed and plated in an RPMI medium supplemented with 20% FBS in the presence or absence of doxycycline (6 µg/ml) and incubated at 37^°^C in 5% CO_2_ for 48 h. Cell viability was measured by trypan blue exclusion assay, as previously described (32). Cell lines CLBL (47) and 17-71 (48, 49) were thawed from frozen stock and analyzed similarly. For statistical analysis, viable cells were reported relative to plating density for both untreated (i.e., control) and treated conditions. To determine if 6 µg/ml of doxycycline was associated with a decreased cell viability, a linear mixed model analysis was performed. Group (i.e., control or treated) was used as a fixed effect within the model with the sample number (i.e., dogs A, B, C, D, CLBL, and 17-71) as a random effect. Transformation of the response variable was performed to meet model assumptions of normality and homogeneous variance. The Wilcoxon signed rank test was also used to evaluate matched pairs for each sample, using mean values from replicates when applicable. Confidence intervals (CIs) were generated from the summary statistics. Significance was defined as *p* < 0.05. Statistical analysis was performed using JMP Pro 13.

## Results

### Clinical Evaluation (Phase II Study and Additional Cohort)

Thirteen client-owned dogs were included in our clinical evaluation (phase II study, *n* = 9; additional cohort, *n* = 4). Eleven dogs were male (nine neutered, two intact) and two were female (one neutered, one intact). There were eight purebred dogs [English Mastiff, Irish Wolfhound, Labrador Retriever, German Shepherd Dog, Weimaraner, Puli, and Rottweiler (*n* = 2)] and five mixed breed dogs. The median age was 6 years (range, 4–13 years). The median body weight was 37 kg (range, 15–66 kg). No dog received corticosteroids within 30 days of enrolment.

At baseline, five dogs were thrombocytopenic (range, 102–168 thou/µl; reference interval, 186–545 thou/µl) and four dogs had presumptive circulating lymphoma cells (*n* = 4; quantified in three dogs; range, 0.2–2.8 thou/µl). Of the nine dogs that had bone marrow cytology evaluated, two dogs were suspected to have a low-level (<5%) lymphoma cell infiltrate. On baseline serum biochemistry, two dogs had grade 2 ALT. Seven other dogs had various biochemical abnormalities that were all only grade 1 in nature. One dog did have renal azotemia and a history of chronic renal disease. Based on the combined results of blood testing and diagnostic imaging, seven dogs had stage V disease, five dogs had stage IV disease, and one dog had stage III disease (Table 1). All 13 dogs were substage a. Cytologically, peripheral lymph node aspirates consisted of a monomorphic population of intermediate to large lymphocytes in all dogs. This was confirmed with flow cytometry. Neoplastic cells were positive for CD45, MHCII, and CD21 in all dogs. Neoplastic cells in some dogs were also positive for CD22 (*n* = 11), CD18 (*n* = 7), CD34 (*n* = 5), and CD20 (*n* = 2). Median forward scatter measurements of the abnormal cells were reported for 11 dogs and ranged from 487 to 623 U (median, 553 U).

**Table 1:** Doxycycline dosage, duration of treatment, and study outcome for individual dogs.

Dog #	Stage^a^	Initial doxycycline dosage (mg/kg, PO BID)	Time in study^b^	Reason for ending study^c^
1	IV	10	2 weeks	24% increase
2	V	10	1 week	Owner withdrawal (19.5% increase)
3	V	10	4 weeks	New site
4	IV	10	1 week	26% increase
5	IV	10	3 weeks	25% increase
6	V	10	8 weeks	Completed study (8% increase)
7	V	7.5	1 week	New site
8	III	7.5	2 weeks	22% increase
9	V	7.5	3 weeks	Owner withdrawal (4% increase)
10	IV	7.5	3 weeks	Owner withdrawal (18% increase)
11	V	7.5	4 weeks	Completed study (21% increase)
12	IV	7.5	3 weeks	Owner withdrawal (1% increase)
13	V	7.5	4 weeks	Completed study (6% increase)

*^a^Stage is presumptive as liver and spleen were only evaluated sonographically and dogs #10–13 did not bone marrow cytology evaluated. All dogs were substage a*.

*^b^The initial study (dogs #1–9) was designed as an 8-week study; the follow-up study (dogs #10–13) was designed as a 4-week study*.

*^c^For dogs #1–9, PD was defined as a new site of disease or an increase in the mean sum LD of ≥20% from best response. For dogs #10–13, PD was defined as a new site of disease or an increase in the mean sum LD of ≥50% from best response*.

The first six dogs enrolled in the phase II study were treated with 10 mg/kg doxycycline PO BID and concurrent metoclopramide. Treatment duration in these six dogs ranged from 1 to 8 weeks (median, 2.5 weeks). No complete responses (CRs) or partial responses (PRs) were observed. One dog had a stable disease (SD) for 6 weeks, four dogs developed PD (median time to progression, 2.5 weeks; range 1–4 weeks), and one dog with SD was removed from the study by the owner after the week 1 evaluation (Table 1). No grade 5 AEs occurred in any of these dogs. Five of six dogs experienced grade 1 (*n* = 3) or grade 2 (*n* = 2) gastrointestinal AEs that included decreased appetite, vomiting, and/or regurgitation. Four dogs were evaluable for hematologic and metabolic/laboratory AEs. Three dogs experienced grade 1 or 2 hematologic AEs. Metabolic/laboratory AEs also occurred in three dogs, with two dogs experiencing grade 3 or grade 4 events. Additional details regarding these AEs are presented in Table 2. Grade 4 ALT, grade 3 ALP, grade 3 AST, grade 1 bilirubin, and grade 1 gastrointestinal AEs developed after 1 week of treatment in the dog with a history of chronic renal disease (dog #6; baseline abnormalities were grade 1 BUN, creatinine, phosphorous, and ALP). At the time of hepatopathy, nodal disease was stable with a 4% decrease in the mean sum LD. Doxycycline and metoclopramide were temporarily discontinued, and famotidine (0.67 mg/kg PO BID) and Denamarin^®^ (425 MG s-adenosylmethionine/35 MG silybin PO every 24 h) were prescribed. At reevaluation 2 weeks later, bilirubin and AST had normalized, and ALT had improved to grade 2; a grade 3 ALP persisted. The mean sum LD had increased by 22% during this 2-week time period. Doxycycline was resumed at a reduced dosage (5 mg/kg PO BID) with concurrent metoclopramide, and these new measurements were considered the baseline or best response. The dog experienced SD for the remainder of the study; ALT eventually normalized, and ALP returned to grade 1. However, progressive azotemia and anemia did develop (≤grade 2), as indicated in Table 2. Ultrasonographic evaluation of the abdomen was repeated, and there continued to be no sonographic evidence of renal lymphoma infiltration. The other dog (dog #1) that developed grade 3 metabolic abnormalities (bilirubin and ALP) suggestive of a hepatopathy had been receiving treatment for 2 weeks and experienced concurrent grade 2 gastrointestinal signs. At baseline, this dog had no metabolic abnormalities, and the liver was normal on sonogram. Doxycycline and metoclopramide were discontinued. Supportive medications including Denamarin^®^ and antiemetics were recommended, as was a corticosteroid due to concurrent PD. The dog’s owner wished to pursue treatment with a homeopathic regimen and declined all recommended medications.

**Table 2:** Adverse events (AEs) in dogs treated with a starting dosage of 10 mg/kg PO BID of doxycycline.

		Gastrointestinal (grade 3/4 not observed)			Hematologic (grade 3/4 not observed)			Metabolic/laboratory				
			
Week	# dogs	Grade 0	Grade 1	Grade 2	Grade 0	Grade 1	Grade 2	Grade 0	Grade 1	Grade 2	Grade 3	Grade 4
1	6^a^	3	3	0	1	0	0	0	1 Cr, P, TB	1 BUN	1 AST, ALP	1 ALT

2	4^b^	2	1	1	2	2 Plt; Hct	0	2	2 BUN, Cr, CK; AST, Alb	1 ALT	2 ALP; ALP, TB	0

3	3^c^	2	0	1	0	3 Plt; Plt; Hct	0	1	1 BUN, Cr, ALT, Am	1 ALT, AST	1 ALP	0

5	1^d^	1	0	0	0	1 Hct	0	0	1 BUN, Cr, Alb, Am	1 ALP	0	0

7	1^d^	1	0	0	0	1 Neu	1 Hct	0	1 Cr, P, ALP, Am	1 BUN	0	0

8	1^e^	1	0	0	0	0	1 Hct	0	1 Cr, P, ALP, Am	1 BUN	0	0

*Number of affected dogs is listed for each week of the study, with specific abnormalities indicated when appropriate. Findings from different dogs are separated by a semicolon. Grade 5 AEs were not observed*.

*Alb, albumin; Am, amylase; ALP, alkaline phosphatase; ALT, alanine aminotransferase; AST, aspartate aminotransferase; BUN, blood urea nitrogen; CK, creatine kinase; Cr, creatinine; Hct, hematocrit; Neu, neutropenia; P, phosphorous; PLT, platelet; TB, total bilirubin*.

*^a^Bloodwork evaluated week 1 in only one dog*.

*^b^One dog temporarily not receiving doxycycline. Three dogs receiving 10 mg/kg PO BID*.

*^c^One dog temporarily not receiving doxycycline. One dog receiving 10 mg kg PO BID and one dog receiving 5 mg/kg PO BID*.

*^d^Dog receiving 5 mg/kg PO BID*.

*^e^Doxycycline discontinued the week prior due to grade 2 anemia and grade 2 increase in BUN*.

The next three dogs (dogs # 7–9) in the phase II study were treated with 7.5 mg/kg PO BID of doxycycline (with concurrent metoclopramide). These three dogs were treated for 1–3 weeks and then came off study due to PD (*n* = 2) and an owner wishing to treat their dog with cytotoxic chemotherapy (*n* = 1) (Table 1). No grade 4 or 5 AEs were observed in these dogs (Table 3). A grade 3 AST was observed in dog #7 concurrent with PD.

**Table 3:** Adverse events (AEs) in dogs treated with a starting dosage of 7.5 mg/kg PO BID of doxycycline.

		Gastrointestinal (grade 3/4 not observed)			Hematologic (grade 3/4 not observed)			Metabolic/laboratory				
			
Week	# dogs	Grade 0	Grade 1	Grade 2	Grade 0	Grade 1	Grade 2	Grade 0	Grade 1	Grade 2	Grade 3	Grade 4
1	7	6	1	0	4	3 Plt; Plt; Hct, Plt	0	4	2 Alb; CK	2 ALT; ALT	1 AST	0

2	6	6	0	0	4	2 Plt; Hct, Plt	0	3	2 AST; ALP, CK	1 ALT	0	0

3	5	5	0	0	2	2 Plt; Hct	1 Plt	2	2 P, CK;AST	0	1 ALT	0

4	2	2	0	0	0	1 Plt	1 Hct	0	2 AST; ALP, Alb, CK	0	1 AST	0

*Number of affected dogs is listed for each week of the study, with specific abnormalities indicated when appropriate. Findings from different dogs are separated by a semicolon. Grade 5 AEs were not observed*.

*Alb, albumin; ALP, alkaline phosphatase; ALT, alanine aminotransferase; AST, aspartate aminotransferase; Ca, hypocalcemia; CK, creatine kinase; Hct, hematocrit; P, phosphorous; PLT, platelet*.

Enrolment in the phase II study was halted after dog #9 was withdrawn. With 9 of 11 dogs enrolled, we knew we would not detect the three responses needed to declare an effect size of 0.3 statistically significant. In order to determine whether a doxycycline dosage of 7.5 mg/kg PO BID had an acceptable AE profile in our target population (i.e., dogs with lymphoma), we evaluated an additional four dogs with multicentric, B-cell lymphoma. This number of dogs was chosen so that when results from all 13 dogs were considered, there were six dogs that had received 7.5 mg/kg of doxycycline PO BID for ≥2 weeks (Table 3). Having observed that dogs that came off study in the phase II evaluation due to PD from progressive nodal enlargement had relatively mild mean sum LD increases of 22–26%, we modified our response criteria such that dogs could remain in study as long as the mean sum LD did not increase to ≥50% from baseline (44). Given the revised objective of tolerability rather than response, the duration of the study was shortened to 4 weeks. These protocol changes were implemented with IACUC approval and revised consent forms. The median treatment duration in this second cohort of four dogs (dogs #10–13) was 3 weeks (range 3–4 weeks). Two dogs were withdrawn by their owners after 3 weeks of treatment in order to start therapy with an oral corticosteroid. All four dogs had SD (range, 1–21% larger than best response) at the time of study completion or withdrawal (Table 1). Gastrointestinal, hematopoietic, and/or metabolic/laboratory AEs were observed in three of these dogs, although no grade 4 or higher AEs were observed (Table 3). Two dogs did experience grade 3 metabolic AEs (ALT and AST).

In summary, no responses (CR or PR) were detected in our clinical evaluation of 13 dogs, and 5 of 13 dogs experienced grade 3 or 4 metabolic/laboratory AEs consistent with hepatopathy. Four dogs with SD (range, 1–19.5% increase in the mean sum LD) were withdrawn from the study by their owners after 1–3 weeks of treatment. The median duration of treatment in the remaining nine dogs was 3 weeks (range 1–8 weeks). At the time of study completion, seven dogs had increases in the mean sum LD (median, 22%; range, 6–25%), and two dogs had developed a new site of disease (Table 1). Because the grade 4 AE occurred in a dog with a history of chronic renal disease, we evaluated baseline renal function parameters in the other four dogs that experienced grade 3 metabolic AEs. In these four dogs, BUN and creatinine concentrations were within the reference intervals, and urine s.g. ranged from 1.012 to 1.048 (median, 1.037; mean, 1.034).

### Doxycycline Concentration in Sera of Treated Dogs

**Figure 1:**
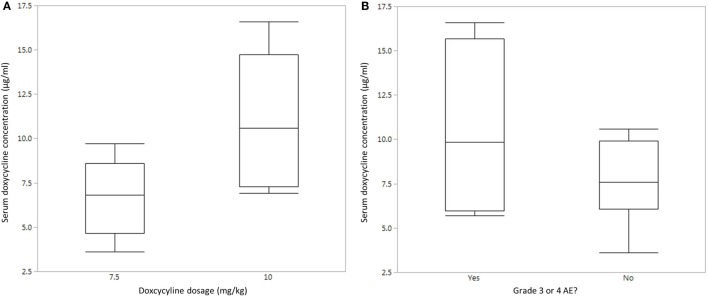
Serum doxycycline concentrations in dogs with B-cell lymphoma. **(A)** Quantile box plot of serum doxycycline concentrations in five dogs that received 7.5 mg/kg PO BID and five dogs that received 10 mg/kg PO BID. **(B)** Quantile box plot of serum doxycycline concentrations in dogs that did (*n* = 4) or did not (*n* = 6) experience a grade 3 or higher adverse event (AE).

In order to determine if lack of clinical response was associated with poor doxycycline absorption in treated dogs, serum doxycycline concentrations were determined. Doxycycline concentrations were measured in five dogs that received 10 mg/kg PO BID (dogs #1–3, 5, and 6) and five dogs that received 7.5 mg/kg PO BID (dogs #7, 8, and 11–13). Results are not reported for the other three dogs as a consequence of no serum sample available for testing (*n* = 1), owner did not administer the appropriate amount of doxycycline (*n* = 1), and poor sample processing (*n* = 1). Doxycycline serum concentration was variable and ranged from 3.6 to 16.6 µg/ml (median, 7.6 µg/ml; mean, 8.8 µg/ml). This is within the range of concentrations that decreased the viability of human DLBCL cells and slowed the growth of xenograft DLBCL tumors in mice (32). Of the 10 dogs that had serum doxycycline concentration determined, four ultimately experienced a grade 3 or higher AE, although not necessarily at the time serum was analyzed. Quantile box plots show that the median serum doxycycline concentration is higher in dogs that received 10 mg/kg PO BID and in dogs that experienced a grade 3 or higher AE (Figures 1A,B). Statistical analysis of these relationships was not performed due to low sample size. [A *post hoc* calculation indicates that power is <50% (α = 0.05) if *n* = 10.] The time of doxycycline administration and serum sample collection was recorded in 7 of 10 dogs, and the interval ranged from 3 to 5 h (median, 3 h; mean, 3.6 h), indicating that our analysis captured peak serum concentration in most dogs.

### *In Vitro* Effect of Doxycycline on the Viability of Canine Lymphoma Cells

**Figure 2:**
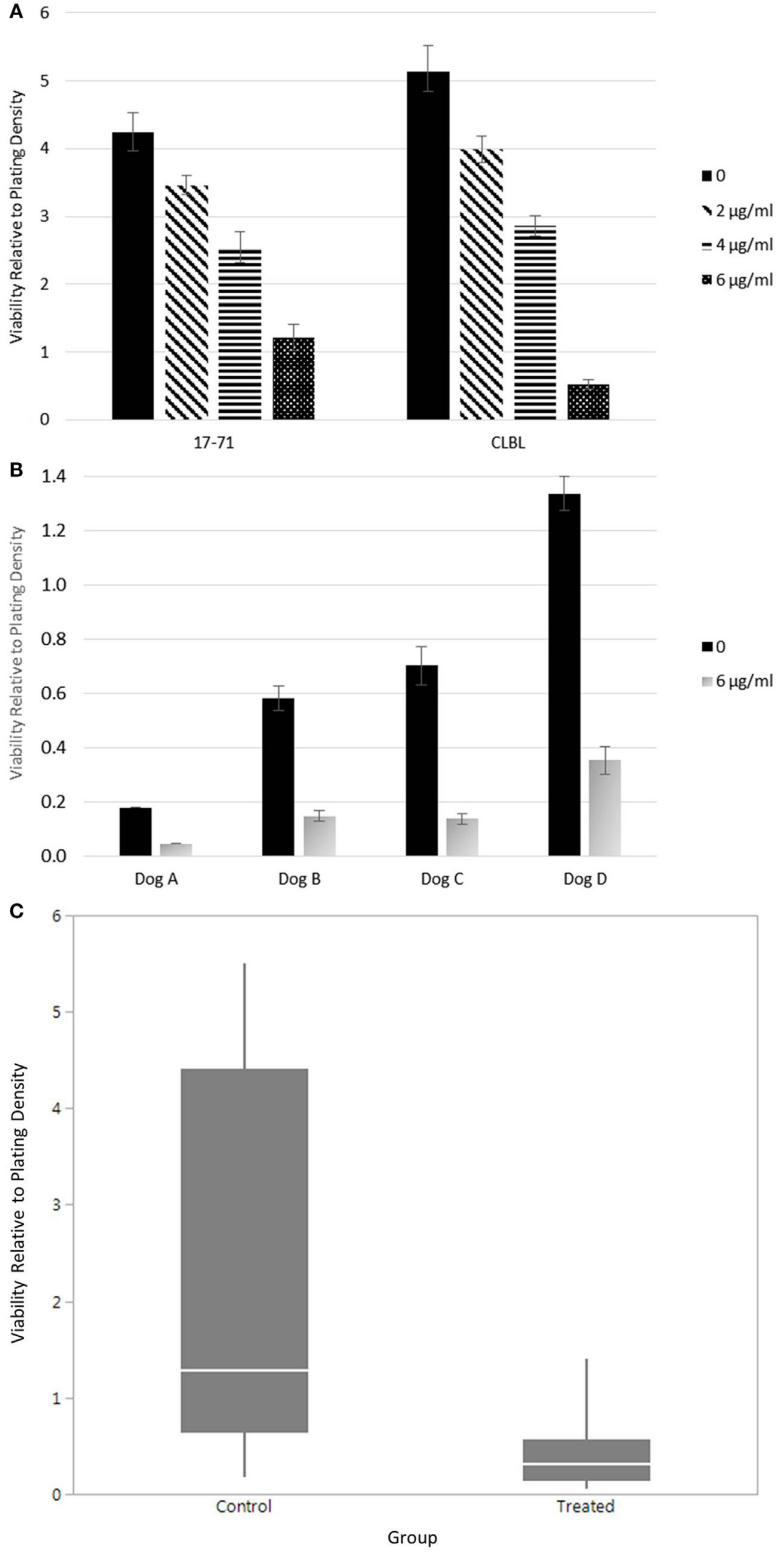
Viability of canine lymphoma cells. **(A)** Relative viability of canine B-cell lymphoma cell lines. Samples were evaluated in triplicate and treated with 48 h of various concentrations of doxycycline. The mean values are presented. Error bars represent standard deviation. **(B)** The relative viability of canine primary B-cell lymphoma cells. The mean values of samples treated with or without doxycycline are presented. Error bars represent standard deviation. Only one replicate was evaluated in Dog A. Dogs B–D were evaluated in triplicate, at minimum. **(C)** Combined statistical analysis for canine B-cell lymphoma primary cells and cell lines. Solid quantile box plot of viability relative to plating density for both control (0 µg/ml) and treated (6 µg/ml doxycycline for 48 h) cells (mixed model analysis, *p* < 0.0001; Wilcoxon signed rank test, *p* = 0.0313).

The lack of clinical response given adequate sera concentrations suggested to us that canine lymphoma cells may not be as susceptible to the same effects of doxycycline as other species, where alterations in cell survival have been noted *in vitro* and in xenograft models (32). To test whether doxycycline exhibits an inhibitory effect on the viability of canine lymphoma cells under *in vitro* culture conditions, as reported for human DLBCL cells, we treated canine B-cell lymphoma cell lines (Figure 2A) and primary cells from lymph nodes of dogs with spontaneously occurring multicentric B-cell lymphoma (Figure 2B) with doxycycline and, using the trypan blue exclusion assay, compared viability to matched untreated control cells. Mixed model analysis (Figure 2C) revealed a significant association between treatment group (i.e., control or treated with 6 µg/ml doxycycline) and relative viability, with control cells having viability equal to the plating density (1.2×), whereas viability in the treated cells was 76% less than the plating density (0.24×) (*p* < 0.0001). For control cells, 95% CI for viability relative to plating density is 1.16–3.18; for treated cells, the 95% CI is 0.22–0.64. Wilcoxon signed rank testing of matched pairs viability also revealed a statistically significant difference between the control and treated (6 µg/ml doxycycline) cells (*p* = 0.0313; control mean, 2.02; treated mean, 0.40; control 95% CI, −0.18–4.21; treated 95% CI, −0.05–0.84). These results suggest that the inhibitory effect of doxycycline, at least *in vitro*, is not limited to human lymphoma cells.

## Discussion

We investigated the clinical response of multicentric, canine B-cell lymphoma to oral doxycycline therapy. Clinical characteristics of the dogs in our study were similar to characteristics described by others (15, 50). We found that a doxycycline concentration of 6 µg/ml was associated with a decreased viability of canine lymphoma cells *in vitro* (Figures 2A–C). However, we did not observe any clinical responses (i.e., CR or PR) in 13 dogs treated with 10 or 7.5 mg/kg doxycycline PO BID despite achieving sera concentrations of >6 µg/ml in at least 8 dogs. We did see SD in one dog for 6 weeks. As it is generally accepted that multicentric canine B-cell lymphoma has a rapid growth rate, achieving SD for this duration may have been a biological improvement for this dog. *Post hoc* power calculations indicate that with a sample size of 8, we had 80% power to call an effect size of 0.4 statistically significant, which would have been realized if 3/8 had responded to treatment. With a sample size of 8, power to call an effect size of 0.3 statistically significant was only 57%, which also would have been realized if 3/8 had responded. Even though we achieved adequate sera concentrations (i.e., >6 µg/ml) in at least eight dogs, it is possible that intracellular doxycycline concentrations were not sufficient to affect the necessary cell-signaling pathways to alter lymphoma cell growth. A limitation of our *in vitro* experiments is that the primary cells were not sorted, and thus samples may have contained a very small number of residual non-neoplastic cells. Additional research is needed to determine if adequate intracellular concentrations of doxycycline can be achieved in dog lymphoma cells *in vivo* and whether appropriate downstream signals are subsequently modified. Our results also may have been affected by the duration of treatment, although eight dogs did receive treatment for ≥3 weeks. As doxycycline would be predicted to alter cell signaling and thus have cytostatic rather than cytotoxic activity, clinical response criteria developed for the evaluation of cytotoxic drugs may not be the ideal way to monitor drug efficacy, and other biomarkers of response and efficacy should be considered in future research (51).

The most concerning AEs in our study were metabolic abnormalities suggestive of hepatopathy, with 5 of 13 dogs (38%) developing grade 3 and/or 4 increases in one or more of bilirubin (*n* = 1), ALT (*n* = 2), ALP (*n* = 2), and AST (*n* = 3). The hepatopathy-related metabolic abnormalities in the dog that experienced a grade 4 ALT either normalized or resolved to grade 1 with a reduced doxycycline dosage, and the dog’s lymphoma remained stable for the remainder of the 8-week study, suggesting that the abnormalities were a direct drug-related AE. In the other four dogs, grade 3 metabolic AEs occurred concurrently with PD, so it remains possible that the abnormalities were secondary to neoplastic infiltration of the liver rather than in direct association with doxycycline, although only one of these dogs had liver abnormalities at baseline. The cytologic or histologic evaluation of the liver should be considered in future research in order to better characterize suspected hepatic pathology. In a retrospective study of 386 dogs where the median doxycycline dosage was 16 mg/kg/day, 39% of dogs experienced increases in ALT and 36% experienced increases in ALP (52). However, the severity and duration of these abnormalities were not reported, so direct comparisons with our study population cannot be made. The dog in our study that experienced a grade 4 metabolic AE did have renal azotemia and a history of chronic renal disease, which may have increased the dog’s likelihood of experiencing an AE. Although there are no reported contraindications to the use of doxycycline in dogs with renal insufficiency, a small amount of drug (generally less than 25%) is excreted in the urine (42, 53, 54). We are unaware of studies evaluating pharmacokinetics specifically in dogs with renal insufficiency; however, doxycycline has been deemed safe in humans with renal insufficiency (55).

Given that the most common side effect of oral doxycycline in dogs is gastrointestinal upset (42), it is not surprising that multiple dogs (6/13, 46%) in our study experienced mild, grade 1 or 2, gastrointestinal AEs. All dogs in our study received concurrent metoclopramide in an effort to minimize gastrointestinal AEs. Interestingly, the use of the antiemetics dimenhydrinate, and metoclopramide was associated with an increased risk of vomiting in a retrospective study of dogs that received doxycycline for a variety of infectious diseases (52). A prospective study to determine whether metoclopramide and other antiemetics alter the risk for gastrointestinal upset in dogs receiving doxycycline is warranted. Six dogs in our study also experienced mild grade 1 or 2 hematologic AEs. In research by others, both healthy, purpose-bred dogs and dogs naturally infected with *Ehrlichia canis* received doxycycline at a dosage of 10 mg/kg PO daily for 28 days and did not develop hematologic or metabolic AEs (56). It is possible that the observed thrombocytopenia and anemia in our study population were secondary to either lymphoma or concurrent disease in some of the affected dogs, as at least two dogs had bone marrow infiltration at the time of staging (bone marrow infiltration was not assessed in two of the affected dogs), and one dog had a history of chronic renal disease.

In our study, a doxycycline dosage of 7.5 mg/kg PO BID was better tolerated than that of 10 mg/kg PO BID, with fewer gastrointestinal AEs and no dog developing a grade 4 metabolic AE. However, 3/7 dogs (43%) still experienced grade 3 metabolic AEs (AST, *n* = 2; ALT, *n* = 1). These dogs were lost to follow-up after the grade 3 metabolic AEs were detected, so we do not know the long-term consequences of those changes. According to VCOG-CTCAE, grade 3 AEs generally are “severe or medically significant but not immediately life-threatening,” whereas grade 4 AEs have “life-threatening consequences,” and medical intervention is considered “urgent” (45). If doxycycline is to be used as an anticancer therapy, continuous administration will likely be required, and therefore one must carefully consider whether persistent metabolic AEs are likely to have adverse clinical consequences in patients. The successful use of doxycycline as an anticancer agent in dogs will require further investigation to understand the incidence and progression of metabolic abnormalities associated with chronic treatment and to determine the dosage required to alter lymphoma cell-signaling events. Dogs with lymphoma that receive doxycycline for antimicrobial purposes should be monitored closely for the development of metabolic abnormalities and/or hepatopathy. Although dogs with other cancers were not included our study population, general precaution and close monitoring are recommended with doxycycline use in these dogs as well.

## Ethics Statement

This study was carried out in accordance with the recommendations of the Cornell University Institutional Animal Care and Use Committee. The protocols (#2014-0022 and #2005-0151) were approved by the Cornell University Institutional Animal Care and Use Committee.

## Author Contributions

The following authors conceived and designed the work: KH, CB, AM-W, CC, and JZ. The following authors acquired, analyzed, or interpreted data for the work: KH, SS, LB, MP, CC, and JZ. The following authors drafted the work and/or revised it critically for important intellectual content: KH, SS, LB, CB, AM-W, MP, CC, and JZ. The following authors gave final approval of the version to be published: KH, SS, LB, CB, AM-W, MP, CC, and JZ. The following authors agree to be accountable for all aspects of the work in ensuring that questions related to the accuracy or integrity of any part of the work are appropriately investigated and resolved: KH, SS, LB, CB, AM-W, MP, CC, and JZ.

## Conflict of Interest Statement

The authors declare that the research was conducted in the absence of any commercial or financial relationships that could be construed as a potential conflict of interest. The reviewer LA and the handling editor declared their shared affiliation.
